# Effects of static stretching and specific warm-up on the repetition performance in upper- and lower-limb exercises in resistance-trained older women

**DOI:** 10.1007/s40520-024-02880-x

**Published:** 2024-12-27

**Authors:** Felipe Lisboa, Witalo Kassiano, Natã Stavinski, Bruna Costa, Gabriel Kunevaliki, Jarlisson Francsuel, Ian Tricoli, Aline Prado, Leticia T. Cyrino, Marcelo A. S. Carneiro, Luis Lima, Pâmela Castro-e-Souza, Edilaine F. Cavalcante, Abdallah Achour Jr., Edilson S. Cyrino

**Affiliations:** Metabolism, Nutrition and Exercise Laboratory, Physical Education and Sport Center, State University of Londrina, Rodovia Celso Garcia, km 380, Londrina, 86057-970 Brazil

**Keywords:** Strength training, Motor performance, Single joint exercises, Aging

## Abstract

**Introduction:**

Preparation methods are often used to improve performance (e.g., number of repetitions) within the resistance training session. However, there is still no consensus in the scientific literature on whether there is a superior preparation method for improving performance, particularly in older adults.

**Methods:**

We compared the effects of preparation by specific warm-up (SW), static stretching (SS), and control condition (CC) on the total number of repetitions in four exercises: leg extension, triceps pushdown, seated leg curl, and preacher curl. Fifty-seven older women (≥ 60 years) performed the experimental protocols (SW and SS) and the CC in a cross-over and counterbalanced design. Following the preparation protocol, the main exercises were performed in two sets until volitional concentric failure, with a two-minute rest interval between sets and 2–3 min between exercises. The main outcome was the total number of repetitions.

**Results:**

The SS improved performance compared to the SW and the CC in the leg extension and seated leg curl resistance exercises. In contrast, the SW impaired performance compared to the SS and CC in the triceps pushdown and preacher curl exercises.

**Conclusion:**

Our results suggest that SS may improve performance in lower-limb exercises, while the SW appears to negatively affect performance in upper-limb exercises in resistance-trained older women.

**Supplementary Information:**

The online version contains supplementary material available at 10.1007/s40520-024-02880-x.

## Introduction

Resistance training (RT) promotes several benefits for muscular performance and health [14, 27]. When planning an RT session, it is common to prescribe preparation methods before the principal sets [12], although the form adopted for this activity is quite different. The most common preparation methods for an RT session are specific warm-up (SW) and static stretching (SS) [12]. The SW is based on performing the same task as the principal exercise, although with a reduced load (30–50% of one-repetition maximum) and an increased number of repetitions (10–15 or more). This strategy promotes an increase in body temperature, heart rate, and oxygen consumption and connects the central nervous system to specific muscle groups [12, 22].

In contrast, SS is a technique that increases musculotendinous and periarticular connective tissue extensibility, contributing to an increased range of motion [13] and possibly improving acute muscle performance since it can improve the efficiency of movements during exercise and increase blood flow to the muscles, increasing the supply of oxygen and nutrients, essential for performance and recovery [4]. Notably, it remains unclear whether such preparation methods result in improved performance, or whether one method is superior to another. Some researchers have investigated the effects of different preparation methods on physical performance measures, particularly acutely before resistance exercises, to ascertain performance-related responses [1, 3, 10, 19, 21].

For instance, Sá et al. [21] reported improvement in leg press performance after SW and SS compared to the control condition (no preparation method). However, SW resulted in a higher number of repetitions in the hack machine squat exercise than the SS. In another report, Ribeiro et al. [19] found no differences in the bench press, squat, and biceps curl exercises performance by comparing control (no preparation method) versus SW. Such findings highlight the uncertainty surrounding this topic. Also, the above-mentioned studies [19, 21] have been conducted in young adults, and less is known about preparation methods in older adults.

Considering that higher RT volume (e.g., performing more repetitions per set) often induces more favorable adaptations in older adults (e.g., reducing metabolic risk and inflammation and inducing greater skeletal muscle mass accretion) [6, 16], identify potential strategies that increase repetition performance would be of great value for this population. Thus, this study aimed to analyze the impact of different preparation methods in the RT session on the number of total repetitions in each exercise. We hypothesized that SW and SS improve performance in total numbers of repetitions compared to a control condition (CC) due to favorable physiological modifications [5, 15, 22]; however, there were no differences between SW and SS.

## Methods

### Experimental approach to the problem

**Fig. 1:**
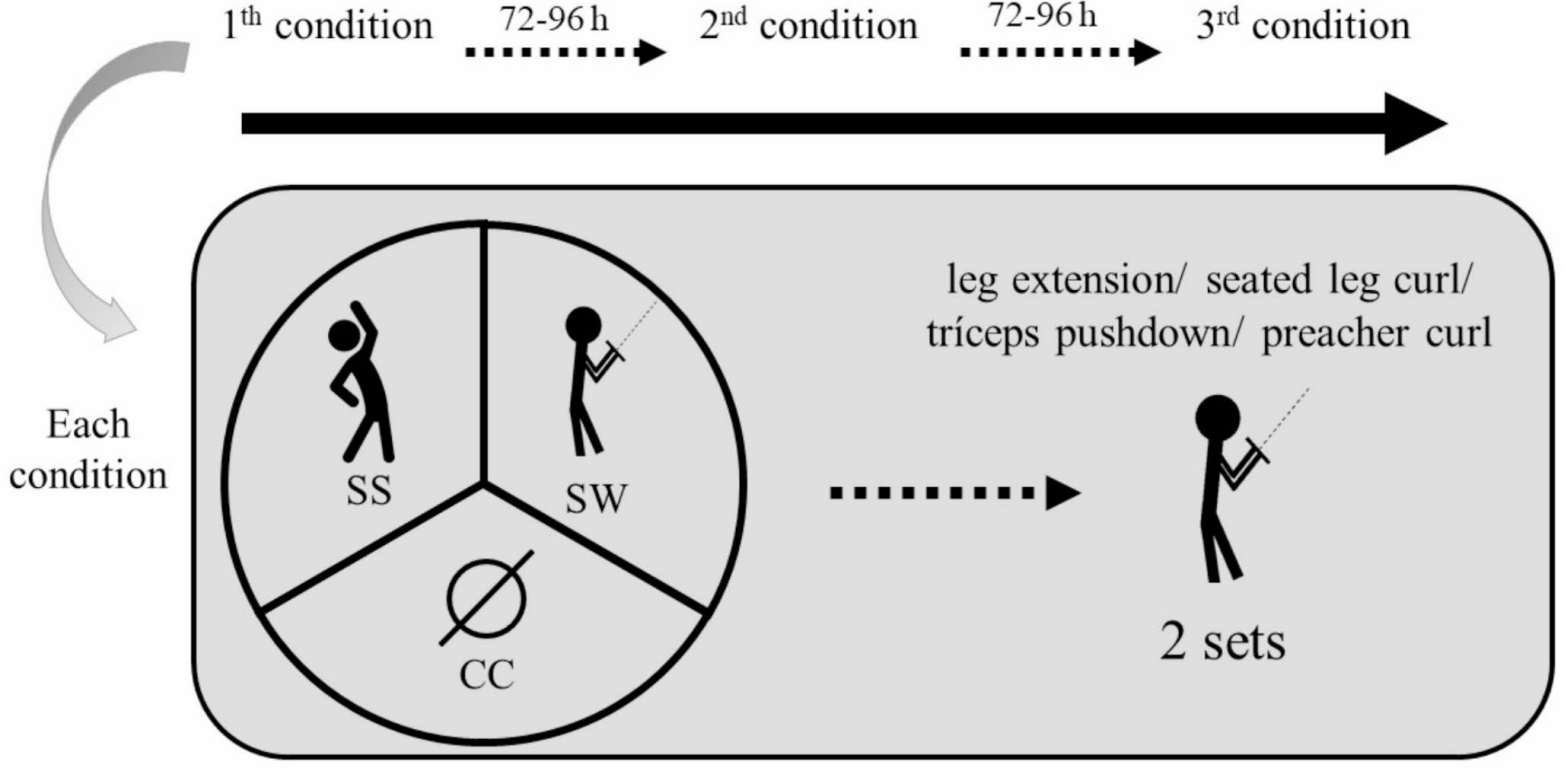
Experimental design. CC = control condition; SW = specific warm-up; SS = static stretching

This study is part of a larger project entitled “Longitudinal Study of Active Aging”, a research project designed to analyze the effects of resistance training on neuromuscular, morphological, metabolic, behavioral, cognitive, and physiological outcomes in older women. A randomized, cross-over, and counterbalanced design was adopted for this study after 24 weeks of RT intervention, as part of a two-year investigation into the impact of RT on cardiac function (NCT06160141). The same resistance exercises (described below) were performed over the 24 weeks of RT that preceded the present acute study. In the first 12 weeks, they performed 10–15 repetitions per set, and in the last 12 weeks, 8–12 repetitions per set. The present study lasted three weeks, where two familiarization sessions were held in the first week, and the other two weeks were used to carry out the protocols, with experimental sessions every 72–96 h. Participants were randomly allocated to three experimental preparation conditions: specific warm-up (SW), static stretching (SS), and control condition (CC). We recorded the total number of repetitions performed in the three experimental conditions in each exercise. All participants performed RT sessions at the same time of day (8–11 a.m.) to minimize possible effects of the circadian cycle on performance. Participants were instructed to maintain their nutritional habits and did not engage in any other physical exercise during this study. A scheme of the experimental design is provided in Fig. 1.

### Subjects

Eighty older women were selected, at the beginning of 2022, through wide dissemination on local radio, newspapers, television, and social media to participate in a progressive RT program for 24 weeks. All participants selected completed health history questionnaires and met the following inclusion criteria: older women, aged ≥ 60 years, physically independent, had no orthopedic conditions that would prevent them from performing the prescribed exercise or exercise testing associated with the study. After the 24 weeks of RT, fifty-seven older women (69.3 ± 5.6 years old, 69.0 ± 12.9 kg, 156.0 ± 5.5 cm) participated in the present acute study. Those participants absent in a specific experimental condition did not have their data computed only for that missing exercise (i.e., available-case analysis). Written informed consent was obtained from all participants after a detailed description of investigation procedures was provided. The investigation was conducted according to the Declaration of Helsinki and was approved by the local University Ethics Committee (1.700.756).

### Experimental resistance training sessions

The resistance training (RT) sessions in this study included eight exercises, performed in the following order: chest press, leg press, seated cable row, leg extension, triceps pushdown, seated leg curl, preacher curl, and seated calf raise. Participants were randomly assigned to start with any exercise in the session, following the above sequence. Each participant started with the same exercise in all experimental conditions, ensuring consistency at the starting point. The preparation methods were manipulated during the leg extension, triceps pushdown, seated leg curl, and preacher curl. This decision was made because they are single-joint exercises with a well-established target muscle, thus facilitating the application of preparation methods. The SW consisted of performing a set lasting 45 s, performing the repetitions within the stipulated time in an execution speed self-selected, with a load of approximately 30–50% of the weight to be used in the 8–12 repetitions in the upcoming resistance exercise. The SS was performed before the first set of each exercise, with unilateral stretching lasting approximately 22 s on each limb, with intensity relative to the first discomfort threshold [4], with the primary muscle group involved in this task as the agonist muscle in the following resistance exercise (e.g., stretching quadriceps before leg extension). In the CC, the participants remained at rest for 45 s before performing in the first set in each exercise. The loads used for the experimental RT sessions were established individually in each exercise, according to the usual load of the previous RT program, equivalent to a zone of 8–12 repetitions. The exercises were performed in two sets until the self-determined maximum repetition (sdRM), that is, the repetitions were interrupted by the participants themselves when they believed they would not be able to perform the next repetition [24]. Participants were instructed to perform each exercise at a tempo of 2/1/0/1 (eccentric, eccentric-concentric transition, concentric, concentric-eccentric transition phases, respectively). The duration of the SW and SS protocols was defined based on the approximate duration of one set (i.e., ~ 4 s per repetition x ~ 12 repetitions = ~ 48 s). A rest interval of two minutes between sets and 2–3 min between exercises was adopted. The total number of repetitions was obtained by the sum of the number of repetitions performed in the first and second principal sets of each analyzed exercise.

### Statistical analysis

The distribution of the data and the homogeneity of the variances were verified using the Shapiro-Wilk and Levene tests, respectively. The effects of the different preparation methods (SW vs. SS vs. CC) on the performance in total number of repetitions were compared using repeated measures analysis of variance (ANOVA), with verification of sphericity by Mauchly’s W test, using Greenhouse-Geisser correction if the assumption of sphericity was violated. Bonferroni’s post hoc test was used to identify differences between experimental protocols when *F*-ratio reached statistical significance. For all statistical analyses, significance was accepted at *P* < 0.05. The effect size between the groups (ES) was calculated using Cohen’s d [9]. An ES < 0.20 was considered trivial, 0.20–0.49 was considered small, 0.50–0.79 was considered moderate, and ≥ 0.80 was considered large. The data were stored and analyzed in Jeffreys’s Amazing Statistics Program (JASP), version 0.14.1 (University of Amsterdam, Amsterdam, NL).

## Results

The results of the comparisons between the different preparation methods applied in the four exercises analyzed are shown in Table 1.

**Table 1:** Number of total repetitions performed in each exercise according to the different experimental conditions

	Experimental Condition			
	Leg extension (*n* = 53)	Seated leg curl (*n* = 52)	Triceps pushdown (*n* = 51)	Preacher curl (*n* = 54)
**Total repetitions**				
CC	25.7 ± 2.6^*^	29.3 ± 4.8^*^	31.0 ± 4.9^†^	28.1 ± 4.3^†^
SW	25.9 ± 4.0^*^	28.1 ± 6.9^*^	28.0 ± 5.7	25.6 ± 5.7
SS	28.2 ± 3.1	36.1 ± 11.9	31.0 ± 5.3^†^	29.0 ± 5.4^†^
**Load (kg)**	27. 7 ± 7.1	36.7 ± 5.9	25.1 ± 4.2	20.2 ± 3.9

*Notes*. CC = control condition; SW = specific warm-up; SS = static stretching. ^*^P < 0.05 vs. SS; †*P* < 0.05 vs. SW. The data are presented as mean and standard deviation

For leg extension, the SS experimental condition resulted in higher mean total repetitions (*P* < 0.05) compared to the SW [mean_diff_ = 2.3 rep (95% CI: 1.0, 3.6), ES = 0.7] and CC [mean_diff_ = 2.5 rep (95% CI: 1.5, 3.4), ES = 0.7]. There was no significant difference (*P* > 0.05) between SW and CC [mean_diff_ = 0.15 rep (95% CI: -1,1, 1.4), ES = 0.04]. Similarly, in the seated leg curl, the SS experimental condition also showed higher mean total repetitions (*P* < 0.05) than SW [mean_diff_ = 8.0 rep (95% CI: 3.9, 12.0), ES = 0.9] and CC [mean_diff_ = 6.8 rep (95% CI: 2.4, 11.2), ES = 0.8]. There was no significant difference *(P* > 0.05) between SW and CC [mean_diff_ = -1.1 rep (95% CI: -3.2, 0.98), ES = -0.1].

For triceps pushdown, SW showed lower mean total repetitions (*P* < 0.05) than SS [mean_diff_ = -2.9 rep (95% CI: -4.9, -0.9), ES = -0.5] and CC [mean_diff_ = -2.9 rep (95% CI: -5.0, -0.7), ES = -0.5]. No significant difference was found between SS and CC (*P* > 0.05) [mean_diff_ = 0.05 rep (95% CI: -1.7, 1.9), ES = 0.01]. In the preacher curl, the SW also showed lower mean total repetitions (*P* < 0.05) compared to SS [mean_diff_ = -3.3 rep (95% CI: -4.7, -1.9), ES = -0.6] and CC [mean_diff_ = -2.4 rep (95% CI: -3.9, -0.8), ES = -0.4]. No significant difference (*P* > 0.05) was revealed between SS and CC [mean_diff_ = 0.9 rep (95% CI: -0.5, 2.3), ES = 0.1].

## Discussion

This investigation compared the effect of different preparation methods on the total number of repetitions in resistance-trained older women. The main findings of this study were that the preparation method influenced muscular performance in lower- and upper-limb resistance exercises. Specifically, we found that the experimental condition SS resulted in a higher number of total repetitions in lower-limb resistance exercises compared to CC and SW, with no difference between CC and SW. For upper limbs, the experimental condition SW resulted in a lower number of total repetitions than CC and SS, with no difference between CC and SS. Therefore, our initial hypothesis was partially confirmed, given that only SS was superior to CC, while SW impaired performance compared to CC and SS. To the best of our knowledge, this is the first study to investigate the effect of different preparation methods on performance in resistance-trained older women.

Despite the often-observed acute increase in the range of motion [4, 15, 25], the use of stretching as a preparation method for performance optimization presents controversial results in the scientific literature. Stretching with a duration ≥ 60 s can be detrimental to performance when compared to shorter durations [7], possibly by increasing stress on the nervous system and hindering muscle activation [15]. For example, such a negative effect does not appear in static stretching lasting approximately 20 s, with intensity relative to the first discomfort threshold [4, 7, 15]. A possible explanation for this phenomenon is that short-duration static stretching does not seem to acutely reduce performance in the total number of repetitions in resistance exercises. For example, Sá et al. [21] found an improvement in performance in the number of total repetitions after an SS protocol in exercises for lower limbs. These findings align with the results of the present study. Notably, there is still no information in the scientific literature that clearly explains the improvement in performance in lower limb exercises provided by short-duration SS. We speculate that our results may be related to increased intramuscular blood flow after static stretching [5] since static stretching generally does not increase muscle temperature significantly, different from dynamic stretching or a light aerobic warm-up that is usually more effective and, therefore, may be related to improved performance [17]. However, these mechanisms were not assessed in our study and remain speculative. Therefore, we believe that static stretching may have contributed to factors that can improve performance without necessarily causing any other damage in return. This potential of static stretching to enhance performance is intriguing and should motivate further exploration. Additional studies are needed to determine the mechanisms that explain such effects of SS on performance during lower-limb resistance exercises.

Our study did not observe improved lower limb performance after SW. As for the upper limbs, SW resulted in decreased repetition performance compared to SS and CC. This result is intriguing because SW is frequently related to improvements in acute performance in resistance exercises [1, 10, 18, 21], although this is not universal [19, 20]. This finding may be associated with the upper-limb characteristics. For example, the voluntary activation level in the upper limbs is usually higher than in the lower limbs [11]. This characteristic suggests that all or most muscle fibers available to perform the task will be more easily activated in the upper limbs than in the lower limbs [11]. If accepted, this hypothesis implies that upper limbs are more stimulated by the same external stimulus and consequently susceptible to faster fatigue than lower limbs since upper limbs appear to have lower muscle endurance than lower limbs for the same relative intensity [2, 23]. Upper limbs also experience more muscle damage than lower limbs in response to a resistance exercise protocol of similar intensity and volume [8].

Unlike previous studies involving SW [1, 10, 18, 19, 21], the participants of this study performed the SW for 45 s, with the relative intensity of 30–50% of the 8–12 repetitions load. Therefore, we do not rule out the possibility that the proposed protocol induced fatigue. As mentioned in the methods, the duration of the SW set was chosen because it was the average duration of the main sets. Furthermore, it is worth noting that the participants were resistance-trained and were used to performing three sets per exercise with a weight corresponding to 8–12 repetitions. Therefore, we hypothesized that this protocol would not result in performance losses compared to other conditions. We would like to note that given that the SW protocol was similar between lower and upper limbs, and there was only impairment to the upper limbs’ performance, our findings suggest that lower intensities or lower duration/number of repetitions of the warm-up set may be a better option for upper limbs in resistance-trained older women. Although reasonable, this hypothesis needs to be tested in future studies. It is noteworthy that the findings of this study can also be related to the investigated population since previous studies conducted with younger populations did not observe loss of performance after the use of SW [10, 19]. Such results may happen, in fact, due to the morphological and functional changes that affect skeletal muscle during the aging process [26]. In this sense, it is necessary to study how such losses can influence muscle endurance and if, in fact, there are differences between the upper and lower muscle groups.

To our knowledge, there are still no studies in the scientific literature that analyzed the effect of different preparation methods on number of repetitions in older adults. Therefore, this factor, together with the design used and the number of participants, is a strength of the present study. However, the lack of monitoring of the intensity of effort and the lack of control of the cadence during the execution of the specific warm-up can be considered limitations of this study. The results of our study are specific to resistance-trained older women, so they should not necessarily be generalized to other populations, including older men and young adults. Finally, future studies are necessary to conduct this experiment chronically, exploring the potential effects of such preparation methods on other exercises and muscles, and investigating the effects of the combination of different preparation methods (e.g., SS plus SW), and also using subjective effort scales, to verify whether any preparation method is capable of reducing the perception of efforts in a fixed number of repetitions, something that may be more common to be seen in practice.

## Conclusions

The method of preparation appears to affect repetition performance during the main sets in resistance-trained older women. More specifically, SS improved the participant’s performance in the leg extension and seated leg curl resistance exercises. Therefore, the prescription of this preparation method should be considered to enhance the performance in the respective lower-limb exercises in resistance-trained older women. On the other hand, SW elicited a decreased performance in the triceps pushdown and preacher curl in comparison to the control no preparation method and SS. Thus, SW may not be the most suitable choice for resistance-trained older women who aim to perform better in the main sets of these upper-limb resistance exercises.

## Electronic supplementary material

Below is the link to the electronic supplementary material.

Supplementary Material 1

## Data Availability

The data is saved and will be made available if requested.
