# Supplementary material for: Effects of static stretching and specific warm-up on the repetition performance in upper- and lower-limb exercises in resistance-trained older women
Source: Aging Clin Exp Res. 2024 Dec 27;37(1):14. doi: 10.1007/s40520-024-02880-x (PMC11671542; doi:10.1007/s40520-024-02880-x)
Supplement: Supplementary file 1 — Supplementary Material 1 [file 40520_2024_2880_MOESM1_ESM.docx]

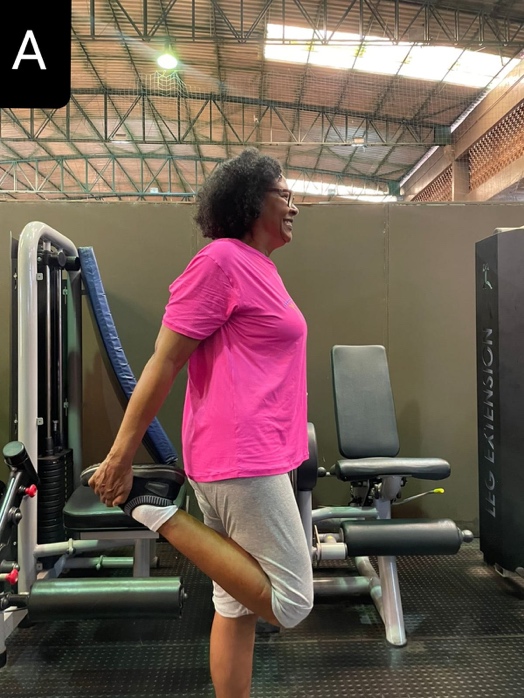

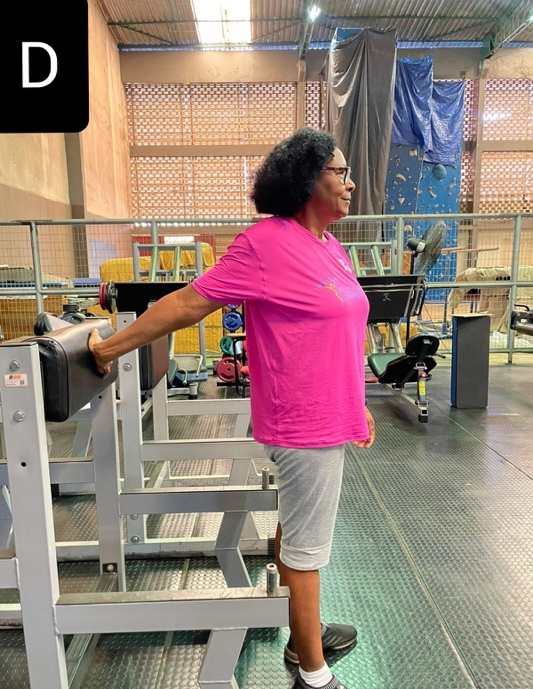

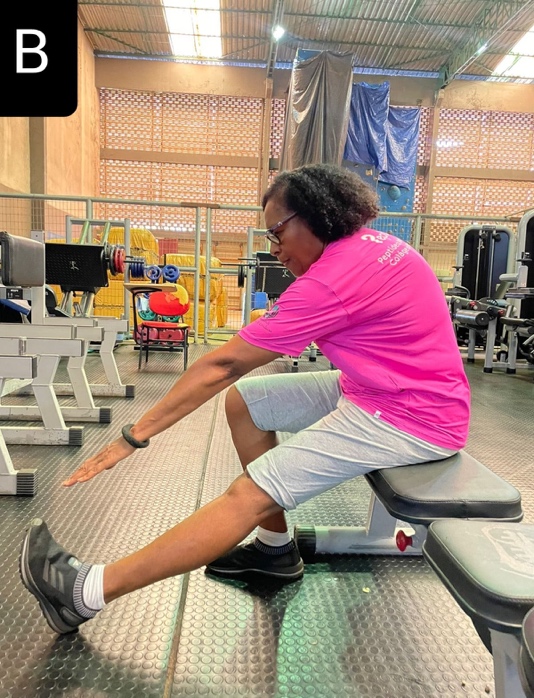


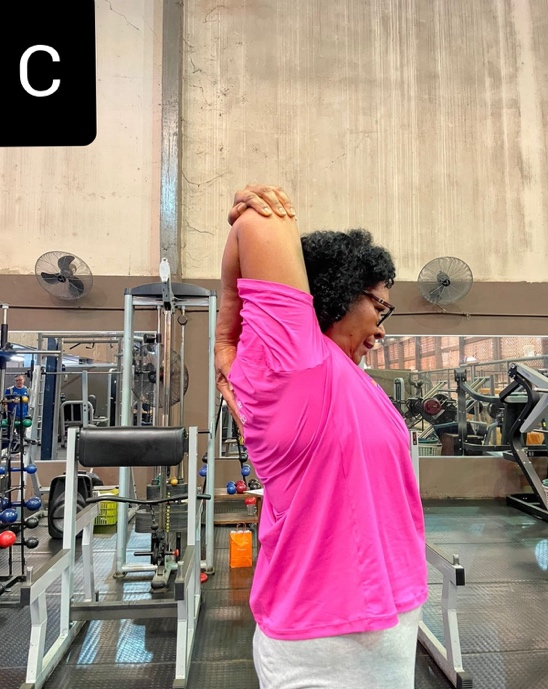


**Supplementary material 1.** Illustration on how the stretching of the quadriceps (A), hamstrings (B), triceps brachii (C), and biceps brachii (D) was done.
